# Poisoning Deaths in a Workshop for Ceremonial Papermaking — China, May 2025

**DOI:** 10.15585/mmwr.mm7530a1

**Published:** 2026-08-06

**Authors:** Zhi Jiang, Siwei Luo, Di Wu, Qi Yang, Fuqin Chen, Jing Chen, Wei Nie, Youming Li, Huihui Liu, Yingxin Pei

**Affiliations:** ^1^Guiyang City Center for Disease Control and Prevention, Guizhou, China; ^2^Chinese Field Epidemiology Training Program, Chinese Center for Disease Control and Prevention & Chinese Academy of Preventive Medicine, Beijing, China; ^3^Songtao County Center for Disease Control and Prevention, Guizhou, China; ^4^Tongren City Center for Disease Control and Prevention, Guizhou, China; ^5^Guizhou Provincial Center for Disease Control and Prevention, Guizhou, China.

**Summary**

**What is already known about this topic?**

Toxic gas production is a recognized hazard in the papermaking process. Deaths from occupation-related exposures have been reported in China, especially in small, unregulated papermaking workshops. Inadequate enforcement of licensing and monitoring requirements increases these risks.

**What is added by this report?**

In May 2025, four employees in an unlicensed southwest China ceremonial papermaking workshop died after being exposed to hydrogen sulfide and other toxic gases in a confined space in the workshop. One woman lost consciousness while cleaning a wastewater tank; three family members, also employees, who attempted to rescue her also died. The workshop had no available personal protective equipment (PPE), atmospheric monitoring, or ventilation.

**What are the implications for public health practice?**

Improved enforcement of workplace safety licensing and hazard identification training and PPE use are essential for reducing occupational exposure risks among workers.

## Abstract

On May 14, 2025, a county-level Chinese Center for Disease Control and Prevention (China CDC) in southwestern China received a report of the sudden death of four employees of a small workshop for ceremonial papermaking, which was operating without required safety and environmental approvals. The first person who died was a woman who lost consciousness while cleaning a wastewater tank. During the next 20 minutes, three of the woman’s family members, also employees, entered the tank to attempt to rescue her. All four persons, including the workshop operator’s wife, the workshop operator’s son, the workshop operator, and the workshop operator’s daughter, died. The county China CDC launched an immediate investigation, and the Intermediate Chinese Field Epidemiology Training Program and provincial and city China CDC staff members conducted a joint, on-site investigation that included identification of cases, analysis of the gas composition, and interviews of residents of the town and physicians to identify the cause of the apparent toxic exposure. Analyses revealed that levels of the highly toxic gases hydrogen sulfide, hydrogen fluoride, and phosphine were several times higher than the maximum allowable concentrations. Clinical signs among the decedents were consistent with poisoning by these gases. Lack of personal protective equipment in a confined space and the presence of toxic gases likely led to the multiple exposures and delayed response by rescue services. Although working in confined spaces with toxic gases is a known exposure risk, these exposures continue to be reported in China and have included deaths in the papermaking industry. These deaths highlight the importance of effective regulations for papermaking workshops and administrative controls, improved supervision, and education for workers.

## Introduction

Ceremonial paper, also known as joss paper, is a traditional item commonly used in Chinese and other East Asian ancestor worship practices. The paper is formed into sheets and effigies that are burned during funerals and festivals to honor deceased ancestors. Ceremonial paper is typically manufactured from low-cost materials, including bamboo pulp, rice paper, or recycled paper, and is often printed with gold or silver foil to resemble currency. During production, the raw material is mechanically pulverized into pulp and then blended into a slurry. Next, water is removed from the slurry; the slurry is then formed, pressed, bleached, and dried into finished joss paper sheets. The pulping and bleaching steps often involve the use of sulfuric acid, sodium sulfate, and sodium hydroxide (*1*). Wastewater from this process, which includes solid particles, is typically discharged into a sedimentation tank for storage, followed by a series of treatments to comply with environmental disposal standards. Hydrogen sulfide gas can be emitted from wastewater through decomposition of sulfur-containing organic compounds and anaerobic sulfate reduction (*2*).

On May 14, 2025, a fatal toxic exposure incident was reported to a county-level Chinese Center for Disease Control and Prevention (China CDC) in southwestern China. Four family members, who were employees of a small workshop for ceremonial papermaking, were found comatose in an underground tank (i.e. a confined space^†^) in the workshop; all subsequently died. The first person, a woman, lost consciousness while cleaning the tank. During the next 20 minutes, three family members, also employees, entered the tank to attempt to rescue her. All four persons, including the son, husband (and workshop operator), and daughter of the woman, died. The county China CDC immediately investigated to determine the cause of the incident and to recommend prevention and control measures to prevent future exposures and deaths. This report describes the investigation and response, including the likely cause of the incident and recommendations for preventing future poisonings.

## Investigation and Findings

### Toxic Exposure Event

On May 14, 2025, the workshop operator’s wife (aged 54 years) and his son (28 years), the workshop operator (57 years), daughter (31 years), and daughter-in-law (28 years) arrived at the processing workshop. Although the daughter was not an employee, she was present on the day of the incident. During the preceding year, the workshop had been conducting production trials; although some paper was produced and stored on-site, none had been sold. The company had a business license (which was obtained online) but did not have a safety production license, an occupational health preassessment, an environmental assessment documentation, or a firefighting inspection certificate, all of which are required for operation. In addition, the workshop lacked any personal protective equipment (PPE), emergency rescue equipment, or protocols for hazard assessment, including testing for toxic gases. This study was reviewed and approved by Guiyang internal review board.^§ ^Yuanbao (Tencent, China), an artificial intelligence assistant, was used during the preparation of this manuscript for language editing and translation support. Authors reviewed and approved all edits.

**FIGURE 1:**
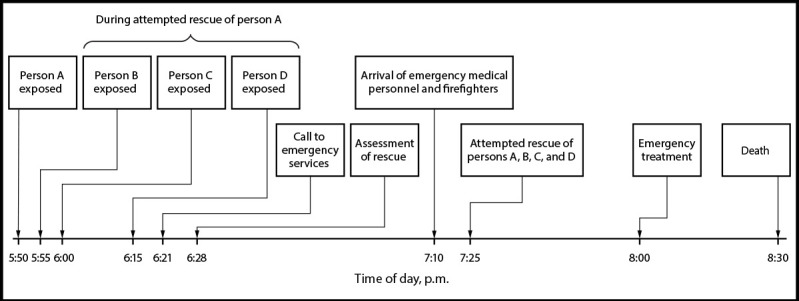
Timeline of events in deadly poisoning of four persons* in a ceremonial papermaking workshop — China, May 14, 2025 * Person A = workshop operator’s wife; person B = workshop operator’s son; person C = workshop operator; and person D = workshop operator’s daughter.

At approximately 5:50 p.m., the operator’s wife descended approximately 7.3 ft (2.2 m) by ladder, without PPE, into the underground tank (Figure 1) to perform the first drainage and sediment removal operation in at least 1 year. Because the tank contained no drainage system, she used an electric water pump to extract and discharge the wastewater. Within minutes, she lost consciousness while in the tank. Her son, the operator (her husband), and her daughter attempted to rescue her, also without using any PPE. These three persons also rapidly lost consciousness in the tank. At 6:21 p.m., the operator’s daughter-in-law asked a neighbor to call medical emergency rescue services. Health center medical staff members from the township (the administrative level below county) arrived within 10 minutes; they detected an unusual odor, and because they also did not have PPE, they called emergency rescue services for additional support. At 7:10 p.m., emergency medical personnel from the county People’s Hospital and firefighters arrived. By 7:25 p.m., all four persons had been removed from the tank by the firefighters. All were cyanotic, unconscious, and without detectable pulse, respiration, blood pressure, or reflexes. All four patients were transported to the county hospital emergency department, where resuscitation efforts were unsuccessful; all were pronounced dead at 8:30 p.m.

### Assessment of the Workshop

**FIGURE 2:**
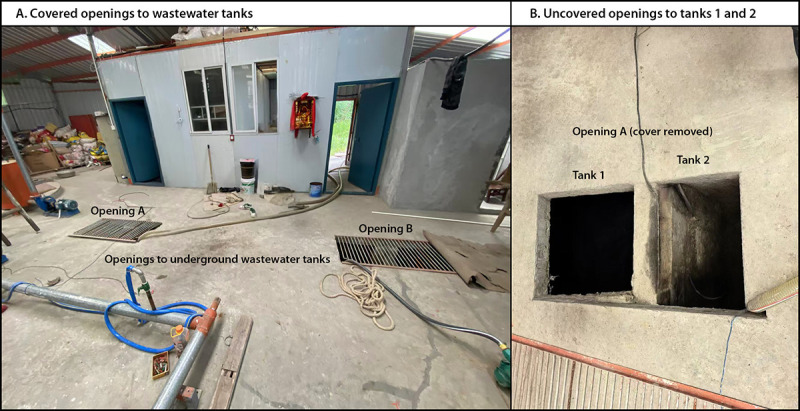
Covered openings to underground wastewater tanks (A)* and uncovered openings to tanks 1 and 2 (B), in a ceremonial papermaking workshop — China, May 14, 2025 * Four wastewater tanks (1, 2, 3, and 4) are located below ground level. Tanks 1 and 2 shared access opening A; tanks 3 and 4 shared access opening B. Tank 2 is the wastewater tank involved in the incident described in this report.

**Workshop characteristics.** The facility consisted of a one-story building that occupied approximately 5,400 sq ft (500 m^2^) and included four underground tanks (Figure 2), each with an approximate volume of 494 cu ft (14 m^3^) and a depth of approximately 7.3 ft (2.2 m). At the time of the investigation, the incident tank contained 6.7 in (17 cm) of wastewater; the other three tanks were nearly full. Two points of entry (approximately 5.4 sq ft [0.5 m^2^] each) provided access to two of the four confined-space underground tanks, all four of which were unventilated and permitted passage, by one person at a time, on a ladder into the tanks. The decedents were standing in the liquid at the time of exposure.

**On-site sampling and testing.** Approximately 22 hours after the incident, occupational health investigators from the provincial China CDC arrived. Wearing PPE (i.e., protective clothing and self-contained breathing apparatuses), they entered the tank to collect gas samples for on-site testing (Table). Testing results showed a mixed exposure ratio^¶^ of hydrogen sulfide, hydrogen fluoride, phosphine, ammonia, and other substances of 106:1, indicating that the combined effect of these substances could create a toxic atmosphere that could immediately result in death.**

**TABLE:** Gas concentration test results from an underground wastewater tank inside a ceremonial papermaking workshop, by detection (sampling) method — China, May 14, 2025

Gas concentration	Detection (sampling) method	
	Rapid detection testing (portable composite gas detector)*	Routine testing (GBZ/T160.33-2004)^†^
**Ammonia**		
Measured concentration	8.1 ppm (5.65 mg/m^3^)	—
Maximum allowable concentration	43.1 ppm (30 mg/m^3^)	—
**Carbon dioxide**		
Measured concentration	2,135 ppm (3,842 mg/m^3^)	—
Allowable short-term concentration	10,000 ppm (18,000 mg/m^3^)	—
**Hydrogen fluoride**		
Measured concentration	10.1 ppm (8.23 mg/m^3^)	—
Maximum allowable concentration	2.4 ppm (2 mg/m^3^)	—
**Hydrogen sulfide**		
Measured concentration	30.5 ppm (42.55 mg/m^3^)	8.4 ppm (11.68 mg/m^3^)
Maximum allowable concentration	7.2 ppm (10 mg/m^3^)	7.2 ppm (10 mg/m^3^)
**Phosphine**		
Measured concentration	20.2 ppm (28.13 mg/m^3^)	—
Maximum allowable concentration	0.22 ppm (0.3 mg/m^3^)	—

**Abbreviation:** ppm = parts per million.

* Rapid detection involves use of portable devices to conduct an on-site analysis at a contaminated site to obtain qualitative, semiquantitative, or quantitative data within a few seconds to a few minutes.

^†^ Routine testing refers to a series of Chinese national occupational standards for determining and controlling the presence of sulfides in workplace air, designed for evaluating chemical hazards in the workplace.

## Public Health Response

Under China’s classification system for public health emergencies, this incident was categorized as a major (level III) public health emergency. The emergency response was initiated within hours and involved emergency management, public health, public security, and environmental protection officials.

### Local Investigation 

Local police immediately closed the workshop to prevent further exposure to the hazardous environment. The county China CDC conducted interviews with all 10 residents of the six households in the village where the workshop was located to gather information about its operations. In addition, a health survey was conducted among the 10 residents to assess the potential impact of toxic gas exposure. The investigation found no evidence of clinical signs or symptoms compatible with exposure among village residents.

### Environmental Investigation

Environmental authorities conducted rapid hydrogen sulfide monitoring inside the tank, at the outlet of the tank, and within the workshop. The tank was identified as the primary source of the gas. Toxic residue was contained and sealed by the local police. Subsequently, the environmental protection department treated the remaining wastewater. Because the residence closest to the workshop was approximately 1/8 mile (200 m) away, and data from the county China CDC household health survey indicated no evidence of exposure to the community, the toxic gas was not expected to have caused any adverse health effects to the surrounding community.

### Regulatory Investigation

China’s regulations mandate that legal operation of a papermaking workshop requires possession of a business license and documentation of an on-site safety inspection by emergency management authorities, an occupational health preassessment by qualified institutions, and an environmental impact assessment approved by environmental authorities. The workshop had a basic business license that was obtained online. The workshop had not yet produced any paper for sale but had operated without the necessary permits for at least 1 year. In response to these findings, recommendations were submitted to government officials to improve the regulatory system to 1) prevent oversight failures and 2) strengthen safety supervision and occupational health training for workers within regulated businesses to reduce their potential for exposure to hazardous substances.

## Discussion

In the year leading up to these four deaths, the workshop had been engaged in intermittent trial production and equipment maintenance, resulting in completely full tanks. Workers pumped out excess wastewater before entry, leaving approximately 6.7 in (17 cm) of liquid at the time of investigation.

Because the tank where the exposure occurred had not been cleaned in at least 1 year, toxic gases had accumulated to critical concentrations. On-site gas sampling suggested that the combined effects of multiple toxic gases likely caused these deaths.

Hydrogen sulfide gas is more dense than air and accumulates in low-lying areas. At high concentrations (>800 parts per million), hydrogen sulfide rapidly causes olfactory fatigue and respiratory failure, resulting in immediate respiratory arrest and shock (*3*,*4*). National data for China indicate that asphyxiating gases, particularly hydrogen sulfide, account for approximately one half of occupation-related poisoning deaths (*5*). Although the use of sulfate in the papermaking processes from this workshop could not be definitively confirmed, standard papermaking practices suggest that sulfate use was likely. In 2013, a similar instance of hydrogen sulfide poisoning was reported in another papermaking business in China, where five persons were poisoned while working without protective equipment or protective measures in place; all five persons survived, although two experienced pulmonary and cerebral edema (*6*).

The detection of hydrogen fluoride and phosphine was unexpected because these gases are typically not associated with the papermaking process. Certain minerals in the tank (e.g., clays, coal, and rocks) might have reacted with alkaline chemicals (e.g., bleach) added during the papermaking process, thereby generating hydrogen fluoride. Phosphine is typically produced through the anaerobic decay and fermentation of organic matter, which might account for its presence in the tank.

Entering confined spaces in a work environment requires a preestablished work plan. These deaths were likely the result of unsafe work practices. The entry of an untrained employee with no PPE into a confined space with no atmospheric monitoring or ventilation, followed by an attempted rescue by untrained employees with no PPE, is similar to a previous incident (2018) in China, during which a rescue attempt resulted in multiple deaths (*7*).

This case highlights a systemic regulatory failure. The workshop lacked mandatory occupational safety permits and environmental impact assessment approvals. Instead, the employees at the workshop relied on self-reporting the permits that they had applied for, rather than written documentation of receipt of mandated permits and completion of required inspections, which allowed noncompliant operations to continue unchecked without implementation of occupational hazard controls (*8*). In addition, human resource limitations, including insufficient staffing, have hampered the ability of relevant authorities to conduct on-site inspections of small workshops, contributing to the regulatory limitations.

Strengthening the safety approval processes administered by emergency management, environmental protection, and health authorities for businesses that produce potentially toxic byproducts can reduce the safety risks faced by workers. In addition, improving employee education and training on safe work practices, occupational hazards, and personal protective measures could reduce the risk for potential toxic exposures (*9*).

### Limitations

The findings in this report are subject to at least two limitations. First, the laboratory analyses of the toxic gases occurred approximately 1 day after the deaths. This delay might have caused an underestimation in the true value of the concentration recorded at the time of the incident, because the tank cover had been removed for nearly 1 day, resulting in sustained ventilation within the tank. Second, because no autopsies were conducted, a documented cause of death could not be obtained. However, the circumstances at the time of death and the clinical findings support the diagnosis of poisoning by toxic gases.

### Implications for Public Health Practice

These poisoning deaths, as well as other documented cases of hydrogen sulfide poisoning deaths among employees and rescuers, highlight the worldwide risk for similar incidents (*10*). These cases expose systemic deficiencies in small business regulation, safety training, ventilation, and atmospheric monitoring. Strengthening licensing, supervision, and operator training can reduce the occurrence of poisoning deaths from toxic gases and overall exposures to toxic gases.

## Footnotes

* These authors contributed equally to this report.

† Confined spaces have limited or restricted means for entry or exit and are not designed for continuous occupancy; they are large enough for workers to enter and perform certain jobs. Confined spaces include but are not limited to tanks, vessels, silos, storage bins, hoppers, vaults, pits, manholes, tunnels, equipment housings, ductwork, and pipelines.

§ Internal review board number MR-52-26-062799.

¶ The mixed exposure ratio is defined as the sum of the ratios of the actual exposure concentrations of various toxic gases in the workplace to their respective occupational exposure limits. This ratio is typically used to assess the combined effect of simultaneous exposure to multiple hazardous gases (when the combined exposure level of multiple toxic gases is below the occupational exposure limit, this value should be ≤1).

** Immediate danger to life and health refers to atmospheres that pose an immediate threat of death or irreversible health effects or that prevent a person from escaping an area.

## References

[1] Liu H. Study on monitoring and analysis method of odor in the kraft pulping process [Chinese]. J Henan Sci Technol 2016;03:144–6.

[2] Chen W, Song PD, Zheng XC, Review of H2S emission and safety issue in wastewater systems [Chinese]. Jishui Paishui 2006;32:15–9.

[3] Andrés CMC, Pérez de la Lastra JM, Andrés Juan C, Plou FJ, Pérez-Lebeña E. Chemistry of hydrogen sulfide-pathological and physiological functions in mammalian cells. Cells 2023;12:2684. 10.3390/cells12232684 38067112 PMC10705518

[4] Sawaya A, Regina AC, Menezes RG. Hydrogen sulfide toxicity. In: StatPearls [internet]. Treasure Island, FL: StatPearls Publishing; May 2, 2024. 32644690

[5] Yuan Y, He Q, Wang D, Characteristics of acute occupational poisoning cases reported in China from 2004 to 2021. Occup Health Emerg Rescue 2023;41:37–42. 10.16369/j.oher.issn.1007-1326.2023.01.009

[7] Zhao LW, Jian TZ, Shi LK, Li YQ, Jian XD, Zhang RH. Investigation of an acute hydrogen sulfide mixture gas poisoning in a confined space [Chinese]. 2022;40:610-2. Chinese J Occup Health 10.3760/cma.j.cn121094-20210808-00388 36052593

[6] Wang Y, Wu G. Investigation of an acute occupational hydrogen sulfide poisoning accident [Chinese]. Chinese J Indust Med 2014;27:160.

[8] Xu X, Ren P, Shi W, Occupational health and safety regulations in China: development process, enforcement challenges, and solutions. Front Public Health 2025;13:1522040. 10.3389/fpubh.2025.1522040 40260161 PMC12009877

[9] Lancia M, Panata L, Tondi V, Carlini L, Bacci M, Rossi R. A fatal work-related poisoning by hydrogen sulfide: report on a case. Am J Forensic Med Pathol 2013;34:315–7. 10.1097/PAF.0000000000000055 24196727

[10] Kage S, Ikeda H, Ikeda N, Tsujita A, Kudo K. Fatal hydrogen sulfide poisoning at a dye works. Leg Med (Tokyo) 2004;6:182–6. 10.1016/j.legalmed.2004.04.004 15231289

